# Hepatitis B Virus X Protein (HBx) Suppresses Transcription Factor EB (TFEB) Resulting in Stabilization of Integrin Beta 1 (ITGB1) in Hepatocellular Carcinoma Cells

**DOI:** 10.3390/cancers13051181

**Published:** 2021-03-09

**Authors:** Chunyan Zhang, Huan Yang, Liwei Pan, Guangfu Zhao, Ruofei Zhang, Tianci Zhang, Zhixiong Xiao, Ying Tong, Yi Zhang, Richard Hu, Stephen J. Pandol, Yuan-Ping Han

**Affiliations:** 1 The Center for Growth, Metabolism and Aging, College of Life Sciences, Sichuan University, Chengdu 610065, China; 2018322040049@stu.scu.edu.cn (C.Z.); yanghuan837@163.com (H.Y.); 2019222040063@stu.scu.edu.cn (L.P.); 2019322040053@stu.scu.edu.cn (G.Z.); 2018222040077@stu.scu.edu.cn (R.Z.); 2017222040084@stu.scu.edu.cn (T.Z.); jimzx@scu.edu.cn (Z.X.); tongying@scu.edu.cn (Y.T.); 2 China West Hospital, Sichuan University, Chengdu 610065, China; Zhangyide520@163.com; 3 Olive View-UCLA Medical Center, Los Angeles, CA 90001, USA; richardhu@mednet.ucla.edu; 4 Cedars-Sinai Medical Center, Los Angeles, CA 90001, USA; Stephen.Pandol@cshs.org

**Keywords:** HBx, TFEB, lysosome biogenesis, ITGB1, cysteine protease, hepatocellular carcinoma (HCC)

## Abstract

**Simple Summary:**

Worldwide, there are more than 800,000 liver cancer patients each year, with more than 700,000 annual deaths. Chronic infection of hepatitis B virus (HBV) contributes to about 70% of the liver cancers. HBV-encoded regulatory protein hepatitis B virus X protein (HBx) is well known for its pleiotropic roles in oncogenesis, in part through promoting viral replication, as well as hijacking host signal transduction pathways. In this study, we observed that lysosomal components and its transcription factor EB (TFEB) were downregulated in hepatocellular carcinoma (HCC) patients. Here, we uncovered a potential mechanism showing HBx-induced oncogenesis may be mediated by downregulation of TFEB, leading to malignant dissemination.

**Abstract:**

Hepatitis B virus (HBV) infection is a major etiological risk for the incidence of hepatocellular carcinoma (HCC), and HBV X protein (HBx) is essential for oncogenic transformation. It is not known that if HBx can sabotage the lysosomal system for transformation and tumorigenesis, or its mechanism if it does have an effect. Examining clinical data, we observed that the downregulation of lysosomal components and transcription factor EB (TFEB) was associated with a poor prognosis of HCC patients. In HCC cells, we found that expression of HBx suppressed TFEB, impaired biogenesis of autophagic-lysosome, and promoted cellular dissemination. HBx mediated downregulation of TFEB led to impairment of autophagic/lysosomal biogenesis and flux, and consequently, accumulation of integrin beta 1 (ITGB1) for motility of HCC cells. Conversely, TFEB, in a steady-state condition, through induction of lysosomal biogenesis restrained ITGB1 levels and limited mobility of HCC cells. Specifically, overexpression of TFEB upregulated and activated the cysteine proteases including cathepsin L (CTSL) to degrade ITGB1. Conversely, expression of cystatin A (CSTA) or cystatin B (CSTB), the cellular inhibitors of lysosomal cysteine proteinases, spared ITGB1 from degradation and promoted dissemination of HCC cells. Taken together, this study suggests a potential mechanism for HBV-mediated malignancy, showing that HBx mediated downregulation of TFEB leads to accumulation of ITGB1 for HCC cell migration.

## 1. Introduction

Liver cancer is the fourth leading cause of cancer-related death worldwide [1]. Hepatocellular carcinoma (HCC) comprises more than 90% of human liver cancers [2]. Risk factors for HCC include aflatoxin exposure, alcoholism, immune-related liver diseases, obesity, and viral infections with hepatitis B virus (HBV) or hepatitis C virus (HCV). Globally, approximately 350 million people are chronically infected with HBV, and 20–30% of chronically HBV-infected individuals may develop cirrhosis and HCC [3]. About 70% of HCC cases are related to HBV infection in developing countries [4]. Regulatory HBV X protein (HBx) is critical for HBV replication and cellular transformation [5,6]. HBx can induce the genetic changes of tumor suppressor genes and oncogenes in liver cancer and can also cause epigenetic aberrations [7,8]. Several studies have shown that HBx interacts with p53 and disrupts its transcription function [9,10]. In HBx transgenic mouse model, occurrence of HCC was significantly increased [11,12]. Despite extensive research, the potential mechanism by which HBx promotes HCC development has not been fully elucidated.

Lysosome, once regarded as a terminal degradative organelle, has been rediscovered for its roles in controlling metabolism, cellular adhesion, migration, and extracellular matrix degradation [13]. Lysosomal dysfunction plays important roles in the development of a wide range of human cancers [14,15]. Lysosomes are contributing precursors for macromolecular synthesis, and therefore important for adaptation to nutrient stress in cancer cells, whereas lysosome-related autophagy plays a critical role in the progression of some tumors. Studies have found that mosaic deletion of Atg5 or liver-specific absence of Atg7 in mice led to multiple liver tumors, suggesting that autophagy plays an important role for suppressing liver tumors [16]. In a genetic study, ectopic expression of the Kras gene in Drosophila was not sufficient for tumor growth and metastasis, but additional lysosomal dysfunction was critically needed, showing that lysosomal flux may delay the malignant transformation [17]. Importantly, through interacting with V-ATPase and impairing the transport to lysosomes, HBx can impair lysosomal acidification and maturation [18]. Thus, these lines of evidence indicate that cellular lysosomal dysfunction or lysosomal stress may promote tumor growth and invasion.

As a member of the MiF family, transcription factor EB (TFEB) is critical for autolysosome biogenesis [19]. Impairment of TFEB-mediated lysosomal biogenesis and autophagy promoted chronic ethanol-induced liver injury and steatosis in mice [20]. The function of TFEB in tumor prognosis is seemingly controversial. For instance, it has been suggested that TFEB was associated with anabolic pathways in pancreatic ductal adenocarcinomas (PDAC) cells [21]. On the contrary, the expression level of TFEB in colorectal cancer has been shown to be significantly low as than that in normal tissues [22]. Knockdown of TFEB in macrophages has dramatically increased tumor growth with enhanced infiltration of M2-like macrophages and angiogenesis of tumors [23]. Thus far, the role of TFEB and its association with HBx and HCC remains unclear.

As a major cysteine proteinase in lysosome, cathepsin L (CTSL) knockout was found to exacerbate tumor growth in the epidermis of the mice expressing human papillomavirus-derived oncogene K14-HPV16 [24]. Importantly, selective knockout of Ctsl in myeloid cells (LysM-Cre/Ctsl^−/−^) of transgenic mice with mammary tumor virus (MMTV)-polyoma middle T (PyMT) led to additional lung metastasis [25]. Cystatin A (CSTA) and cystatin B (CSTB) are endogenous inhibitors of cathepsins B, H, and L. It has been reported that CSTB is often increased in HCC patient’s liver tissue and serum [26,27]. Thus, a steady-state of the autophagy-lysosome pathway is essential for cellular homeostasis, and its malfunction is associated with tumorigenesis.

Integrins (ITGs) are cell surface adhesion molecules that mediate cell adhesion, contractility, and motility [28,29,30]. Endocytosis and recycling of integrin dysfunction has been reported to promote the spread and migration of epithelial cells [31]. In the same way, knockout of integrin beta 1 (ITGB1) has been shown to impair breast cancer initiation. In addition, in a transgenic mouse model, expression of ITGB1 resulted in increased development of papilloma and lymph node metastases [32]. Alteration of integrin β1(ITGB1) spatial distribution and temporal expression have been frequently observed in multiple cancers including HCC [33]. As compared with surrounding non-cancerous tissues, ITGB1 has been shown to be overexpressed in HCC [34]. Similarly, its high expression has often been found in several different HCC cell lines [35].

To understand the function of TFEB in liver cancer, we retrieved expression levels of TFEB and lysosome components in TCGA database and the Kaplan–Meier (KM) plotter. We found that TFEB and the key components of lysosome were downregulated in liver cancer. In HCC cell lines, we demonstrated that HBx could downregulate TFEB and impair lysosomal biogenesis and flux. Cellular TFEB, through activation of autolysosome flux and induction of CTSL, could induce ITGB1 degradation, which consequently suppressed the cancer cell migration.

## 2. Results

### 2.1. Downregulation of Transcription Factor EB (TFEB) and Lysosomal Components Are Related to Liver Cancer Incidence and Poor Prognosis

**Figure 1:**
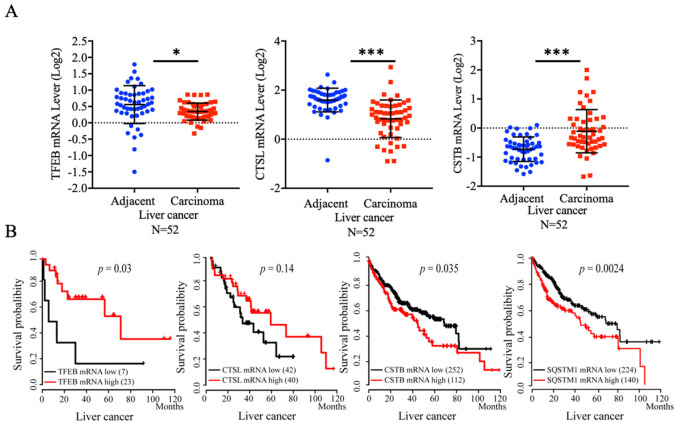
Downregulation of transcription factor EB (TFEB) and lysosomal components are related to hepatocellular carcinoma (HCC) incidence and poor prognosis. (**A**) The mRNA levels of TFEB, cathepsin L (CTSL) or cystatin B (CSTB) in liver cancer samples were retrieved from The Cancer Genome Atlas (TCGA) database; (**B**) The Kaplan–Meier plots of the overall survival (OS) of human liver cancer patients were stratified for TFEB, p62/SQSTM1, CTSL or CSTB mRNA expression levels, and the log-rank test. *P* values were shown. * *p* < 0.05 and *** *p* < 0.001.

Initially, we searched The Cancer Genome Atlas (TCGA) database and Kaplan–Meier plotter (www.kmplotter.com, accessed on 2 January 2021) for the clinical relevance of lysosomal biogenesis in liver cancer. Indeed, we found that low expression of TFEB was statistically associated with the incidence of liver cancer, as shown in Figure 1A. Consistently, low expression of CTSL, a prominent lysosomal proteinase that is controlled by TFEB, was also significantly associated with the incidence of liver cancer. The survival rate of patients with low expression of TFEB and CTSL was related to a poor prognosis (Figure 1B). Conversely, high expression of CSTB, an endogenous inhibitor of lysosomal cathepsins, and p62/SQSTM1, was related to poor prognosis of liver cancer. We also examined other TFEB regulated genes which have coordinated lysosomal expression and regulation (CLEAR) element in the gene promoters. As compared with the adjacent area, the expression of other auto-lysosomal components including lysosomal lipase (LIPA), lysosomal membrane component (LAPTM4A), as well as cathepsin S (CTSS) and cathepsin Z (CTSZ) (two lysosomal cysteine proteinases), was significantly decreased in the carcinoma area (Figure S1). Taken together, these results suggest that the impeded lysosomal biogenesis is associated with the development and malignancy of liver cancer.

### 2.2. Hepatitis B Virus X Protein (HBx) Suppresses TFEB and Impairs Lysosomal Biogenesis to Promote Cancer Cell Migration

**Figure 2:**
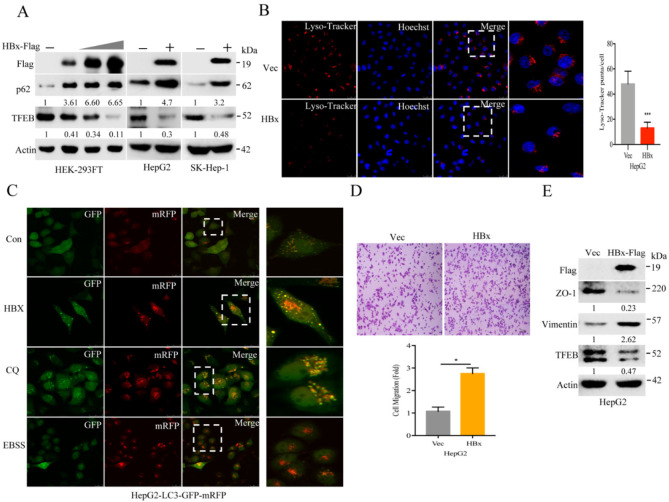
Hepatitis B virus X protein (HBx) suppresses the expression of TFEB and impairs lysosome biogenesis. (**A**) HepG2 or SK-Hep-1 cells stably expressing HBx and HEK-293FT were transfected with vector control or 0.5, 1, or 2 μg of HBx plasmid, followed by Western blotting analysis. (**B**) Lyso-Tracker staining assay. HepG2 cells stably expressing vector (Vec) or HBx were treated with 100 nM Lyso-Tracker for lysosomes followed by confocal microscopy analysis. The experiment was repeated three times and five visual fields were counted for statistics. (**C**) HepG2 cells stably expressing LC3-tandem-mRFP-GFP were further infected with HBx lentivirus or treated with EBSS medium for 6 h, or chloroquine at 50 μM, for 12 h, followed by confocal microscopy analysis. (**D**) HepG2 cells stably expressing vector (Vec) or HBx-Flag were subjected to transwell assay. The experiment was repeated three times and five visual fields were counted for statistics; (**E**) HepG2 cells stably expressing vector control (Vec) or HBx-Flag were subjected to Western blotting analysis for ZO-1, vimentin, TFEB, and actin. For (B) and (C), scale bar = 25 μm; for (D), scale bar = 100 μm. * *p* < 0.05 and *** *p* < 0.001.

HBx is known to be a strong viral oncoprotein for cellular transformation and HBV replication. In two HCC cell lines as well as HEK-293FT cells, we found that expression of HBx could downregulate TFEB along with p62 accumulation (Figure 2A), indicating that HBV suppressed lysosomal flux. HBx was able to inhibit lysosomal biogenesis as visualized by fluorescent Lyso-Tracker staining (Figure 2B and Figure S2 in Supplementary Materials). Expression of HBx decreased the number and size of lysosome puncta. Autophagic flux was measured through expression of tandem fluorescence-tagged LC3 (tf-LC3), which is a probe for determining auto-lysosomal acidification [36]. As shown in Figure 2C and Figure S2B, expression of HBx failed to quench green fluorescence LC3 and accumulation of red-colored auto-lysosomal puncta, suggesting impairment of auto-lysosomal flux. This was further validated by a treatment with chloroquine, an autolysosome inhibitor, showing accumulation of yellow-colored puncta in the cells. Conversely, the increased auto-lysosomal flux was evident by EBSS treatment, which amino acids are depleted, leading to inactivate mTOR pathway and activation of auto-lysosomal flux.

It is known that HBx, through activation of STAT5 beta or c-Src, for epithelial–mesenchymal transition (EMT), can promote motility and invasiveness in HCC cells [37,38]. Here, we validated the notion, showing increased motility of HepG2 cells by expression of HBx (see Figure 2D). Moreover, expression of HBx significantly downregulated ZO-1 along with accumulation of vimentin. Taken together, these results suggest a potential mechanism showing that HBx suppresses TFEB and impairs lysosomal biogenesis to promote cell migration.

### 2.3. TFEB Inhibits Cell Migration through Downregulation of Integrin β1

**Figure 3:**
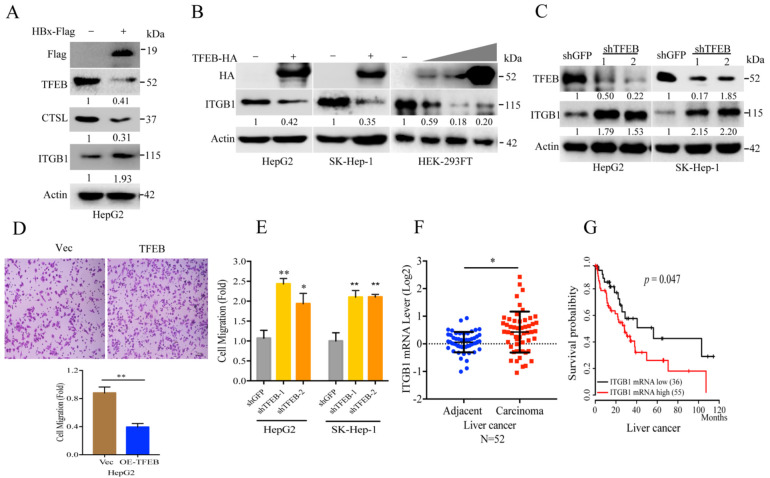
TFEB inhibits cell migration through downregulation of integrin beta 1. (**A**) HepG2 cells stably expressing vector (Vec) or HBx-Flag were subjected to Western blotting analysis for TFEB, CTSL, integrin beta 1 (ITGB1) and actin. (**B**) HepG2 or SK-Hep-1 cells stably expressing TFEB and HEK-293FT transfected with vector control or 0.5, 1, or 2 μg of TFEB-HA plasmid, were subjected to Western blotting analyses for ITGB1. (**C**) HepG2 or SK-Hep-1 cells were infected by lentivirus to knockdown TFEB, followed by Western blotting analysis for ITGB1. (**D**) HepG2 cells stably expressing TFEB were subjected to transwell assay. The experiment was repeated four times and five visual fields were collected for analysis. (**E**) HepG2 or SK-Hep-1 cells with knockdown of TFEB were subjected to transwell assay. The experiment was repeated three times and five visual fields were collected for analysis. (**F**) The mRNA levels of ITGB1 by the liver cancer samples were retrieved from TCGA database. (**G**) The Kaplan–Meier plots of overall survival (OS) of human liver cancer patients were stratified by ITGB1 mRNA expression levels, the log-rank test. For (D), scale bar = 100 μm. *p* Values were showed. * *p* < 0.05 and ** *p* < 0.01.

Thus, we explored the potential mechanism of the HBx mediated auto-lysosomal stress and cell motility of HCC. Cell-stromal anchorage mediated by integrins is critical for cancer cell activation, awaking cell dormancy and motility [39]. Here, we found that expression of HBx downregulated TFEB and simultaneously increased ITGB1 expression (see Figure 3A) which indicates a potential degradation control. Therefore, we examined TFEB-mediated integrin beta 1 (ITGB1) turnover by testing three cell lines and found that overexpression of TFEB could thoroughly downregulate ITGB1, indicating that lysosome may mediate ITGB1 turnover (Figure 3B). In contrast, knockdown of TFEB restored ITGB1 level (see Figure 3C). Expression of TFEB significantly decreased the migration ability of HepG2 (see Figure 3D). On the contrary, knockdown of TFEB increased motility of HCC cells (see Figure 3E). We also retrieved the mRNA level of ITGB1 from TCGA database and the survival curve in KM plotter. Here, we found that high level of ITGB1 in liver cancer patients was related to a poor prognosis (see Figure 3F,G). Thus, these results indicate a potential mechanism showing that TFEB inhibits cell migration through downregulation of ITGB1.

### 2.4. Cellular Turnover of ITGB1 Is Controlled by TFEB and Mediated by Autolysosomal Flux

**Figure 4:**
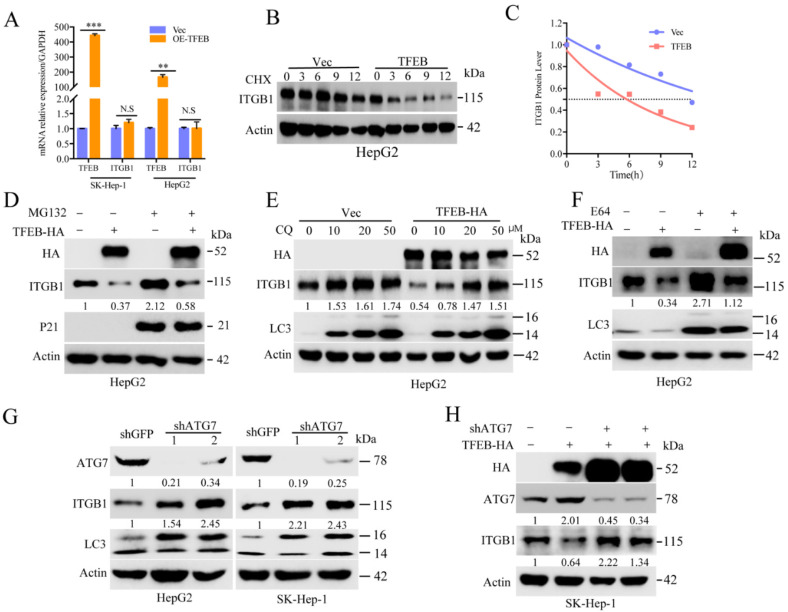
TFEB-induced auto-lysosomal pathway promotes integrin beta 1 degradation. (**A**) The ITGB1 mRNA levels of HepG2 or SK-Hep-1 cells stably expressing TFEB were determined by RT-qPCR analysis. The experiment was repeated three times; (**B**,**C**) HepG2 cells expressing TFEB or Vec were treated with 20 μg/mL cycloheximide (CHX) for the indicated time interval, followed by Western blotting analysis. The ITGB1 levels were quantified by densitometry and the turnover rate was presented; (**D**–**F**) HepG2 cells expressing TFEB or Vec were treated with 10 μM MG132 for 8 h, or a gradient of concentration chloroquine for 12 h, or 50 μM E64 for 24 h as indicated, followed by Western blotting analysis; (**G**) HepG2 or SK-Hep-1 cells were infected by lentivirus to ATG7, followed by Western blotting analysis; (**H**) HepG2 or SK-Hep-1 cells stably expressing TFEB were infected with lentivirus encoding shATG7, and then were subjected to Western blotting analysis for ITGB1. ** *p* < 0.01 and *** *p* < 0.001. N.S. = no significance.

The results mentioned above indicated that a steady state of cellular TFEB could restrain cancer cell motility in part via acceleration of the turnover of ITGB1. To further exam this, we measured the mRNA levels of ITGB1 in HepG2 and SK-Hep-1 cells stably expressing TFEB. The results showed that there was no significant change in the mRNA levels of ITGB1 in the condition of TFEB overexpression (see Figure 4A), suggesting the regulation may be regulated at the protein level. The protein turnover rate of ITGB1 was determined by a treatment with cycloheximide (CHX), an inhibitor for ribosome. As shown, turnover of ITGB1 was significantly accelerated in the cells overexpressing TFEB (see Figure 4B,C). Next, we probed the pathways for TFEB-mediated ITGB1 turnover using proteinase inhibitors. As shown, MG132, a proteasome inhibitor, slightly increased ITGB1, while chloroquine significantly rescued ITGB1, indicating autolysosome-mediated turnover of ITGB1 (see Figure 4D,E). Moreover E-64, a potent irreversible inhibitor against lysosomal cysteine proteases, could also thoroughly restore ITGB1 (see Figure 4F and Figure S3A). ATG5 and ATG7 are key factors involved in phagophore formation and auto-lysosomal flux. An shRNA-based knockdown of ATG7 or ATG5 could restore the expression of ITGB1, in agreement with attenuation of autophagic flux (see Figure 4G,H and Figure S3B). Taken together, these results demonstrate that cellular turnover of ITGB1 is controlled by TFEB-induced auto-lysosomal machinery.

### 2.5. TFEB-Mediated Upregulation of Lysosomal CTSL Promotes ITGB1 Degradation

**Figure 5:**
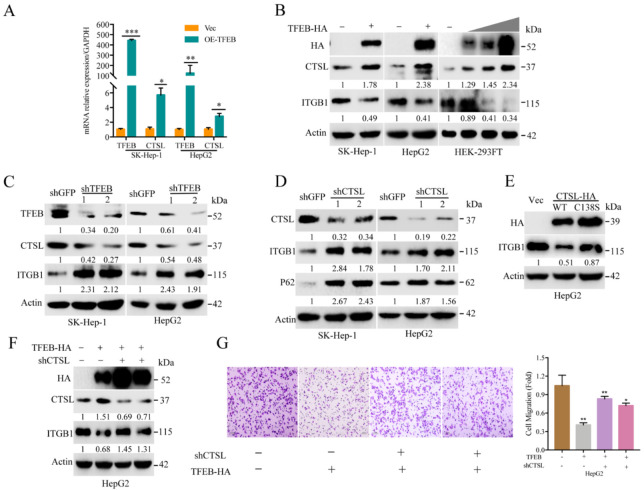
TFEB-mediated upregulation of lysosomal CTSL promotes ITGB1 degradation. (**A**) The mRNA level of CTSL and TFEB of HepG2 or SK-Hep-1 cells expressing TFEB were determined by RT-qPCR analysis. The experiment was repeated three times for statistics; (**B**) HepG2 or SK-Hep-1 cells stably expressing TFEB or HEK-293FT transfected with vector control or 0.5, 1, or 2 μg of TFEB-HA plasmid were subjected to Western blotting analysis; (**C**) HepG2 or SK-Hep-1 cells were infected by lentivirus to knockdown TFEB, followed by Western blotting analysis; (**D**) HepG2 or SK-Hep-1 cells with knockdown of CTSL were subjected to Western blotting analysis; (**E**) HepG2 cells expressing either CTSL-HA or CTSL-HA (C138S) were subjected to Western blotting analysis; (**F**,**G**) HepG2-TFEB-HA or SK-Hep-1-TFEB-HA cells were infected by lentivirus to knockdown CTSL, followed by Western blotting (**F**) or Transwell assay (**G**). Scale bar = 100 μm. The experiment was repeated three times and five visual fields were counted for each condition. For (**D**), scale bar = 100 μm. * *p* < 0.05, ** *p* < 0.01, and *** *p* < 0.001. N.S. = no significance.

As mentioned above, TFEB could promote ITGB1 turnover through the lysosomal pathway. Next, we investigated the specific lysosomal enzyme for ITGB1 degradation. As shown, in response to overexpression of TFEB, the number and size of lysosomal puncta were abundantly increased (Figure S4). Furthermore, we found that overexpression of TFEB could substantially upregulate the mRNA level of CTSL (see Figure 5A). At the protein level, overexpression of TFEB led to upregulation of CTSL, which consequently promoted degradation of ITGB1 (see Figure 5B). Likewise, knocking down of TFEB resulted in suppression of CTSL and, consequently, restoration of ITGB1 (see Figure 5C). Moreover, knocking down CTSL led to attenuation of auto-lysosomal flux, showing accumulation of p62/SQSTM1 and ITGB1 (see Figure 5D). Furthermore, we expressed a catalytically null variant of CTSL (C138S mutant) and compared that with the wild type. As shown, expression of the catalytically inactive CTSL variant (C138S) failed to downregulate ITGB1 (see Figure 5E). Next, we investigated whether CTSL could inhibit motility of HCC cancer cells. As shown in Figure 5F,G, shRNA-based knockdown of CTSL restored ITGB1 and promoted cell migration. Taken together, these results demonstrate that TFEB-induced ITGB1 degradation can restrain the mobility of cancer cells in part via the CTSL mediated lysosomal degradation.

### 2.6. Endogenous Inhibitors of Lysosomal Cysteine Proteinases Control the Degradation Flux of ITGB1

**Figure 6:**
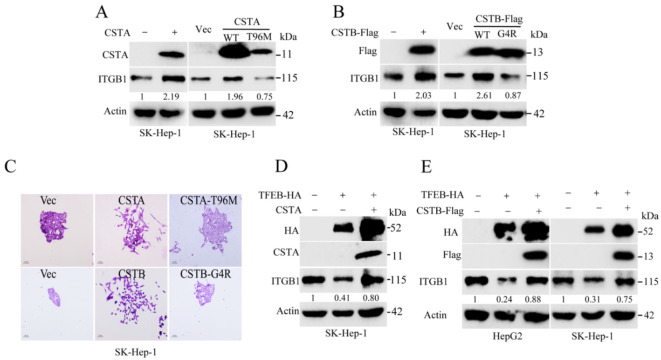
Lysosomal proteinase inhibitors CSTA and CSTB suppress the turnover of ITGB1. (**A**) SK-Hep-1 cells expressing either CSTA or CSTA^T96M^ were subjected to Western blotting analysis. (**B**) SK-Hep-1 cells expressing CSTB-Flag or CSTB^G4R^-Flag were subjected to Western blotting analysis. (**C**) SK-Hep-1 cells expressing CSTA or CSTA^T96M^ or CSTB-Flag or CSTB^G4R^-Flag were subjected to colony formation analysis. (**D**) SK-Hep-1 cells expressing TFEB were infected with lentivirus encoding CSTA, then subjected to Western blotting analysis. (**E**) HepG2 or SK-Hep-1 cell expressing TFEB were infected with lentivirus encoding CSTB-Flag, then subjected to Western blotting analysis.

Cystatins are cellular reversible inhibitors of the lysosomal and cysteine proteases. We noticed that ectopic expression of CSTA or CSTB, inhibitors of cathepsins B, H, and L, could actually restore ITGB1 expression (see Figure 6A,B and Figure S5A,B). Overexpression of CSTA or CSTB promoted mobility of SK-Hep-1 cells (see Figure 6C). Conversely, expression of cystatin mutants, CSTA^T96M^ or CSTB^G4R^, showed no significant impact on ITGB1 turnover and cell migration. Overexpression of TFEB decreased the protein level of ITGB1, which could rescue by expression of CSTA or CSTB, as shown in Figure 6D,E. Therefore, the cellular turnover of ITGB1 is controlled by the balance of lysosomal proteinases and endogenous inhibitors. These results further validate the notion that TFEB-induced lysosomal biogenesis can determine the turnover of ITGB1.

## 3. Discussion

HBV is a major pathogen for hepatocellular carcinoma (HCC) [40]. As an oncoprotein, HBx is essential for initiating and maintaining viral replication after infection [41]. Multiple cellular machineries can be hijacked by HBx for the viral proliferation and host cell transformation. In this study, we found HBx could downregulate TFEB, a master transcription factor for autophagy-lysosome biogenesis. The mechanism for the HBx-mediated downregulation of TFEB is a subject of ongoing investigation. Here, we noticed that HBx-mediated downregulation of TFEB led to accumulation of ITGB1. Integrins play important roles in cell adhesion, migration, proliferation, and phenotypic determination. In HCC, the expression of ITGB1 in patient tissues was significantly increased [33]. A recent study found that integrin beta 1 could inhibit the TGF-β mediated activation of hepatic stellate cell (HSC) [42]. HSC activation and cirrhosis are essential for the initiation and progression of HCC. EMT is often associated with cancer cell activation and dissemination. Indeed, we noticed that HBx in hepatocytes could downregulate ZO-1 and also promote the accumulation of ITGB1. It is known that the WNT/beta-catenin pathway is frequently activated in HCC [43,44].

In agreement with HBx-mediated downregulation of TFEB, we found the impediment of lysosomal biogenesis, showing downregulation of lysosomal proteinase CTSL and accumulation of p62/SQSTM1, and impaired autophagic flux. Increased integrin anchorage, focal adhesion, and signaling are known for promoting motility of cancer cells [45,46]. As a consequence of the HBx-mediated downregulation of TFEB, we noticed that ITGB1 was accumulated in the HCC cells. Thus, we focused on investigating how cellular TFEB controls ITGB1 turnover. In particular, we confirmed that overexpression of TFEB could promote biogenesis of lysosome in HCC cells. As demonstrated in this study, cellular turnover of ITGB1 was mostly mediated by autophagic-lysosomal flux rather than the proteasome pathway. Furthermore, we found that CTSL was responsible for ITGB1 degradation, which was counteracted by CTSA and CTSB, the cellular inhibitors for the lysosomal cathepsins. Moreover, we found downregulation of TFEB and accumulation of ITGB1 were related to increased cellular motility. Importantly, in the clinical data, low expression of TFEB was statistically associated with the incidence of liver cancer, indicating that TFEB may prevent liver cancer from metastasis. Consistent with this study, downregulation of TFEB increased colorectal cancer risk [22] and promoted melanoma metastasis [47]. However, upregulation of TFEB was related to accelerated PDAC cell proliferation [21]. In general, TFEB may play dual roles in different cancers, depending on the context and niche.

Autophagy pathway has been reported to be involve in cell migration [48]. Snail, which is an important transcription factor regulating EMT, could be degraded though the autolysosome pathway, inhibiting tumor metastasis [49]. As a downstream gene of TFEB, CTSH could degrade class-II HDACs, known for tumorigenesis and malignancy [50]. Consistently, increased autophagic activity inhibited HCC cell lines migration [51].

However, the functional relationship between TFEB and cell motility remains unclear. We demonstrated that knockdown of TFEB increased stabilization of ITGB1 and promoted cell migration of HCC. Taken together, this work revealed that TFEB suppressed HCC cells migration through CTSL mediated degradation of ITGB1, by which HBx promoted the HCC migration.

## 4. Materials and Methods

### 4.1. Reagents and Antibodies

Chloroquine (C6628), polybrene (TR-1003), and puromycin (540222) were from Sigma-Aldrich (Taufkirchen, Germany). LysoTracker (L7528) and Lip3000 (L3000008) were from Thermo Fisher (Waltham, MA, USA). Cycloheximide (S7418) and MG132 (S2619) were from Selleck (Houston, TX, USA). The qPCR Mix (E096-01B) were from Novoprotein (Shanghai, China). Antibodies used in this study were as follows: beta-actin (RLM3028) from Ruiyingbio (Suzhou, China); human influenza hemagglutinin, HA-tag (#3724) from Cell Signaling Technology (Danvers, MA, USA); TFEB (YT4631) and CTSL (YT5124) from ImmunoWay Biotechnology Company (Plano, TX, USA); GAPDH (200306), p62/Sqstm1 (380612), and LC3 (306019) from Zen BioScience (Chengdu, China ); ITGB1 (SC-8978) from Santa Cruz BioTechnology (SantaCruz, CA, USA); CSTA (HPA001031) and FLAG (F1804) from Sigma (Taufkirchen, Germany); ATG5 (CY5766) and ATG7 (CY5658) from Abways Biotechnology (Shanghai, China).

### 4.2. Plasmids

Plasmid pcDNA3.1-TFEB (#99955) was purchased from Addgene (Watertown, MA, USA). Plasmid FLAG-HBx-PMV, FLAG-CSTA-PMV, or FLAG-CSTB-PMV were generated in Wuxi Qinglan Biotech Co. Ltd. (Wuxi, China). TFEB, CSTA, CSTB, and HBx fragments were constructed into PLVX-Puro or PLVX-BSD by double enzyme digestion and confirmed by sequencing analysis. Plasmid pLVX-Puro-CTSL^WT^ or pLVX-Puro-CTSL^C138S^ were stored in this laboratory. The variants of CSTA^T96M^ or CSTB^G4R^ were generated by PCR. Tandem monomeric mRFP-GFP-tagged LC3 was a kind gift from Dr. Ying Tong. The shRNAs were cloned into pLKO.1. The sequence information of the primers used for shRNA or variants is described in Supplementary Materials Table S1.

### 4.3. Cell Culture and Construction of Stable Cell Lines

HepG2 and HEK-293FT were obtained from ATCC (American Tissue Culture Collection). Hepatocellular carcinoma cell line SK-Hep-1 was kindly provided by Dr. Yong Peng, Sichuan University. SK-Hep-1, HepG2, and HEK-293FT were cultured in DMEM (Hyclone, Logan, UT, USA) supplemented with 10% fetal bovine serum and 1% penicillin/streptomycin. All cells were grown at 37 °C in humidified incubators with 5% CO2. Lentiviral particles were produced by transiently transfecting HEK293FT cells with lentiviral vectors together with pMD2G and psPAX2 using Lipo3000. After 48 h, lentiviral particles were harvested from supernatant, and filtered through a 0.45 μm membrane. Target cells were incubated with lentiviral particles together with polybrene for 24 h. Puromycin (2.5 μg/mL) was added to medium to select for resistant cells.

### 4.4. Western Blotting Analysis

Cells were lysed with RIPA buffer as reported earlier [52]. The concentration of proteins was measured using the BCA protein assay kit (Thermo Scientific, Waltham, MA, USA). Total protein lysates (20 μg) were loaded for 10% SDS-PAGE, followed by transfer to PVDF membranes. The blots were incubated with the corresponding primary antibodies in TBS-T buffer, at 4 °C for overnight, and then incubated with appropriate secondary antibody conjugated with horseradish peroxidase (HRP). Proteins were visualized with ECL blotting substrate.

### 4.5. Reverse Transcription Quantitative PCR (RT-qPCR) Analysis

Total RNA was extracted with Trizol (Biosharp, Hefei, China), followed by reverse transcription using reverse transcription kit (Roche, Basel, Switzerland). The RT-qPCR assays were performed as reported earlier [53]. Primer sequences were as follows: ITGB1-F, TGGACAATGTCACCTGGAAA; ITGB1-R, AGCTCCTTGTAAACAGGCTGAA; CTSL-F, AGGAGAGCAGTGTGGGAGAA; CTSL-R, ATCTGGGGGCCTCATAAAAC; GAPDH-F, AAAATGGCAGTGCGTTTAG; GAPDH-R, TTTGAAGGCAGTCTGTCGTA; TFEB-F, CCAGAAGCGAGAGCTCACAGAT; TFEB-R, TGTGATTGTCTTTCTTCTGCCG. The results were analyzed using 2^−∆∆Ct^ method.

### 4.6. Transwell Cell Migration Assay

The assay was performed in 8.0 μm pore size transwell inserts (Corning, NY, USA). Cells were suspended in serum-free medium and seeded into the upper chambers, as a chemoattractant the lower chamber contained 500 μL complete medium. Cells were incubated for 12 h at 37 °C in a humidified incubator with 5% CO_2_. Next, the chambers were fixed with cold 100% methanol and stained with 0.1% crystal violet for 20 min, the non-migration cells on the inside of the chamber were carefully removed with cotton swabs, and then photographed with light microscopy and the data was analyzed by Image J software (NIH, Bethesda, MD, USA).

### 4.7. Cell Morphology

For cell morphology, 1 × 10^3^ cells/well were plated in six-well plates, grown for 7~10 days, fixed with 100% methanol, stained with 0.1% crystal violet, and then photographed by light microscope.

### 4.8. Cellular Protein Turnover Assay

Cells overexpressing TFEB were treated with or without cycloheximide at a final concentration of 20 μg/mL and collected at the indicated time points. The ITGB1 protein levels were checked by Western blotting analysis. Intensities of bands for ITGB1 were quantified with Image Lab (Bio-Rad) software and the data was further normalized with β-actin levels.

### 4.9. Tandem Fluorescence-Tagged LC3 Probe

Cells stably expressing LC3-tandem-mRFP-GFP or stably expressing LC3-tandem-mRFP-GFP and HBx were cultured on coverslips for 24 h, and then treated with indicated condition as shown, photographed by confocal microscopy.

### 4.10. Lysosome Staining

Cells overexpressing TFEB or HBx were cultured on coverslips for 24 h, and then treated with 100 nM LysoTracker (Thermo Fisher, Waltham, MA, USA) for 1 h to label the lysosomes. The nuclei were labeled with Hoechst (Sigma-Aldrich, Taufkirchen, Germany). And the coverslips were analyzed by confocal microscopy.

### 4.11. Gene Expression in HCC, Retrieved from Public Data Bank

The expression analysis of TFEB, ITGB1, CTSL, ITGB1, and CSTB at their mRNA levels in HCC was retrieved from TCGA database. The patient’s survival curve was analyzed from KM plotter (www.kmplotter.com, accessed on 2 January 2021).

### 4.12. Statistical Analysis

Each experiment was conducted at least three times, and the results were expressed with error bars which denote SD. The differences between two groups were assessed using a two-tailed Student’s t-test. One-way analysis of variance statistics was used to compare the means of three groups or more, followed by a Tukey’s multiple comparison test. * *p* < 0.05, ** *p* < 0.01, and *** *p* < 0.001. N.S. = no significance. The *p* values less than 0.05 were considered to be significant.

## 5. Conclusions

In this study, we identified a potential mechanism showing HBx-induced tumorigenesis through impairment of lysosomal biogenesis. In particular, our work suggests that HBx mediated cell migration may be regulated through downregulation of TFEB, leading to accumulation of ITGB1. Conversely, cellular TFEB may suppress HCC through CTSL-mediated degradation of ITGB1. Therefore, this study reveals a potential new mechanism for HBx mediated tumorigenesis of HCC.

## Data Availability

Materials are available on request.

## Supplementary Materials

The following are available online at https://www.mdpi.com/2072-6694/13/5/1181/s1, Figure S1. Expression of LIPA, LAPTM4A, CTSS and CTSZ in the liver of human HCC was retrieved in TCGA database; Figure S2. HBx impaired TFEB induced lysosome biogenesis; Figure S3. TFEB promoted integrin β1 degradation through auto-lysosomal pathway; Figure S4. TFEB promoted lysosomal biogenesis; Figure S5. Cellular turnover of ITGB1 was controlled by lysosomal proteinase inhibitors; Table S1. Sequence information of the primers used for shRNA or variants.

## Abbreviations

HBV, hepatitis B virus; HBx, hepatitis B virus X protein; HCC, hepatocellular carcinoma; TFEB, transcription factor EB; ITGB1, integrin beta 1; CTSL, cathepsin L; ATG*5*, autophagy related homolog 5; ATG*7*, autophagy related homolog 7; CSTA, cystatin A; CSTB, cystatin B; TCGA, The Cancer Genome Atlas; CLEAR, coordinated lysosomal expression and regulation, EMT, epithelial–mesenchymal transition.
