# Correction: Zhang et al. Hepatitis B Virus X Protein (HBx) Suppresses Transcription Factor EB (TFEB) Resulting in Stabilization of Integrin Beta 1 (ITGB1) in Hepatocellular Carcinoma Cells. *Cancers* 2021, *13*, 1181

**DOI:** 10.3390/cancers17010103

**Published:** 2024-12-31

**Authors:** Chunyan Zhang, Huan Yang, Liwei Pan, Guangfu Zhao, Ruofei Zhang, Tianci Zhang, Zhixiong Xiao, Ying Tong, Yi Zhang, Richard Hu, Stephen J. Pandol, Yuan-Ping Han

**Affiliations:** 1 The Center for Growth, Metabolism and Aging, College of Life Sciences, Sichuan University, Chengdu 610065, China; 2018322040049@stu.scu.edu.cn (C.Z.); yanghuan837@163.com (H.Y.); 2019222040063@stu.scu.edu.cn (L.P.); 2019322040053@stu.scu.edu.cn (G.Z.); 2018222040077@stu.scu.edu.cn (R.Z.); 2017222040084@stu.scu.edu.cn (T.Z.); jimzx@scu.edu.cn (Z.X.); tongying@scu.edu.cn (Y.T.); 2 West China Hospital, Sichuan University, Chengdu 610065, China; zhangyide520@163.com; 3 Olive View-UCLA Medical Center, Los Angeles, CA 90001, USA; richardhu@mednet.ucla.edu; 4 Cedars-Sinai Medical Center, Los Angeles, CA 90001, USA; stephen.pandol@cshs.org

In the original publication [1], there was a mistake in Figure 3D as published. Unfortunately, incorrect parts were included within the image during the editing process. The corrected Figure 3 appears below.

The authors apologize for any inconvenience caused and state that the scientific conclusions are unaffected. This correction was approved by the Academic Editor. The original publication has also been updated.

## Figures and Tables

**Figure 3:**
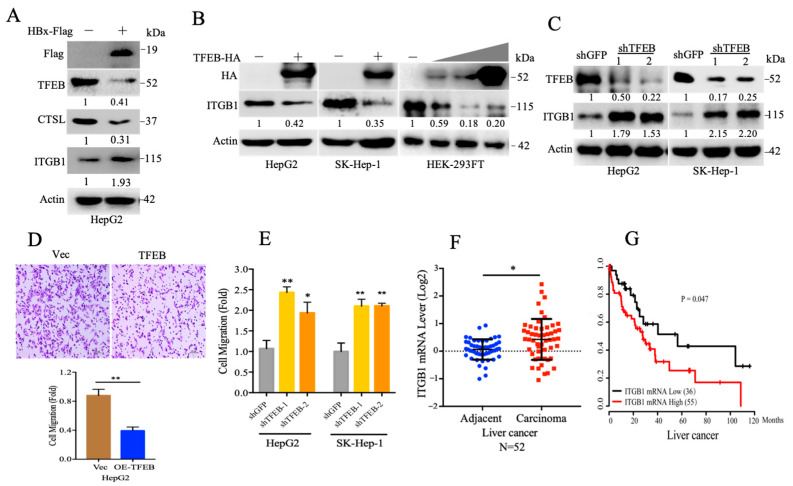
TFEB inhibits cell migration through downregulation of integrin beta 1. (**A**) HepG2 cells stably expressing vector (Vec) or HBx-Flag were subjected to Western blotting analysis for TFEB, CTSL, integrin beta 1 (ITGB1), and actin. (**B**) HepG2 or SK-Hep-1 cells stably expressing TFEB and HEK-293FT transfected with vector control or 0.5, 1, or 2 μg of TFEB-HA plasmid, were subjected to Western blotting analyses for ITGB1. (**C**) HepG2 or SK-Hep-1 cells were infected by lentivirus to knockdown TFEB, followed by Western blotting analysis for ITGB1. (**D**) HepG2 cells stably expressing TFEB were subjected to transwell assay. The experiment was repeated four times, and five visual fields were collected for analysis. (**E**) HepG2 or SK-Hep-1 cells with knockdown of TFEB were subjected to transwell assay. The experiment was repeated three times, and five visual fields were collected for analysis. (**F**) The mRNA levels of ITGB1 by the liver cancer samples were retrieved from TCGA database. (**G**) The Kaplan–Meier plots of overall survival (OS) of human liver cancer patients were stratified by ITGB1 mRNA expression levels, the log-rank test. For (**D**), scale bar = 100 μm. *p* Values were shown. * *p* < 0.05 and ** *p* < 0.01.

## References

[1] Zhang C., Yang H., Pan L., Zhao G., Zhang R., Zhang T., Xiao Z., Tong Y., Zhang Y., Hu R. (2021). Hepatitis B Virus X Protein (HBx) Suppresses Transcription Factor EB (TFEB) Resulting in Stabilization of Integrin Beta 1 (ITGB1) in Hepatocellular Carcinoma Cells. Cancers.

