# Supplementary material for: Hepatitis B Virus X Protein (HBx) Suppresses Transcription Factor EB (TFEB) Resulting in Stabilization of Integrin Beta 1 (ITGB1) in Hepatocellular Carcinoma Cells
Source: Cancers (Basel). 2021 Mar 9;13(5):1181. doi: 10.3390/cancers13051181 (PMC7967237; doi:10.3390/cancers13051181)
Supplement: Supplementary file 1 [file cancers-13-01181-s001.zip › cancers-1114373-supplementary/cancers-1114373-supplementary.pdf]

Supplemental Figures

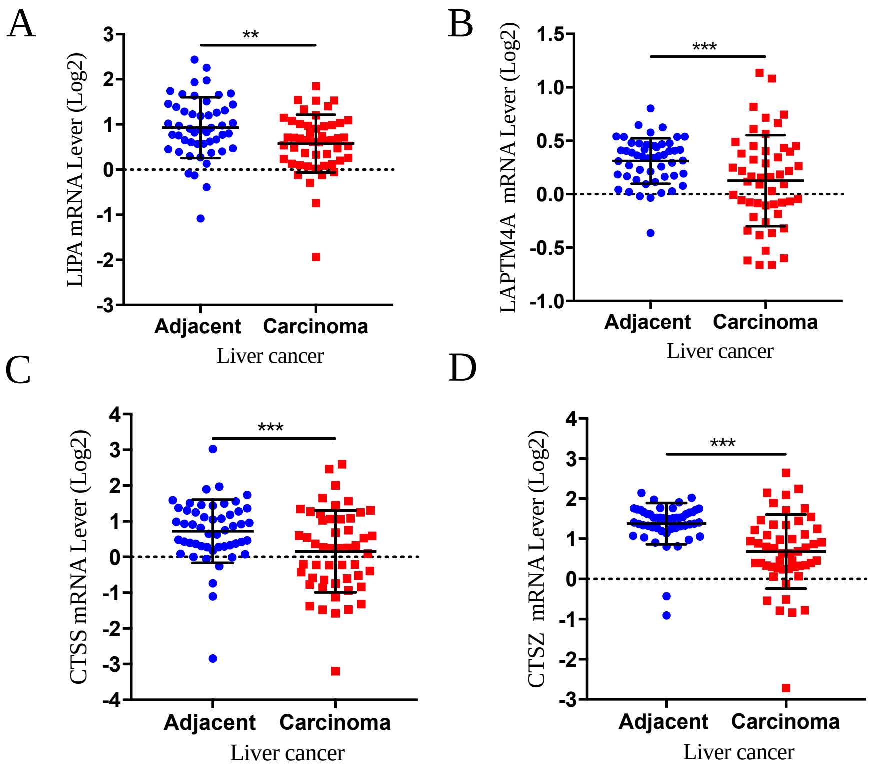

Figure. S1. LIPA, LAPTM4A, CTSS and CTSZ expression were retrieved in human liver cancers. **(A-D)** mRNA levels of LIPA, LAPTM4A, CTSS and CTSZ in adjacent or liver cancer samples were analyzed using TCGA database. ** $p<0.01$; *** $p<0.001$

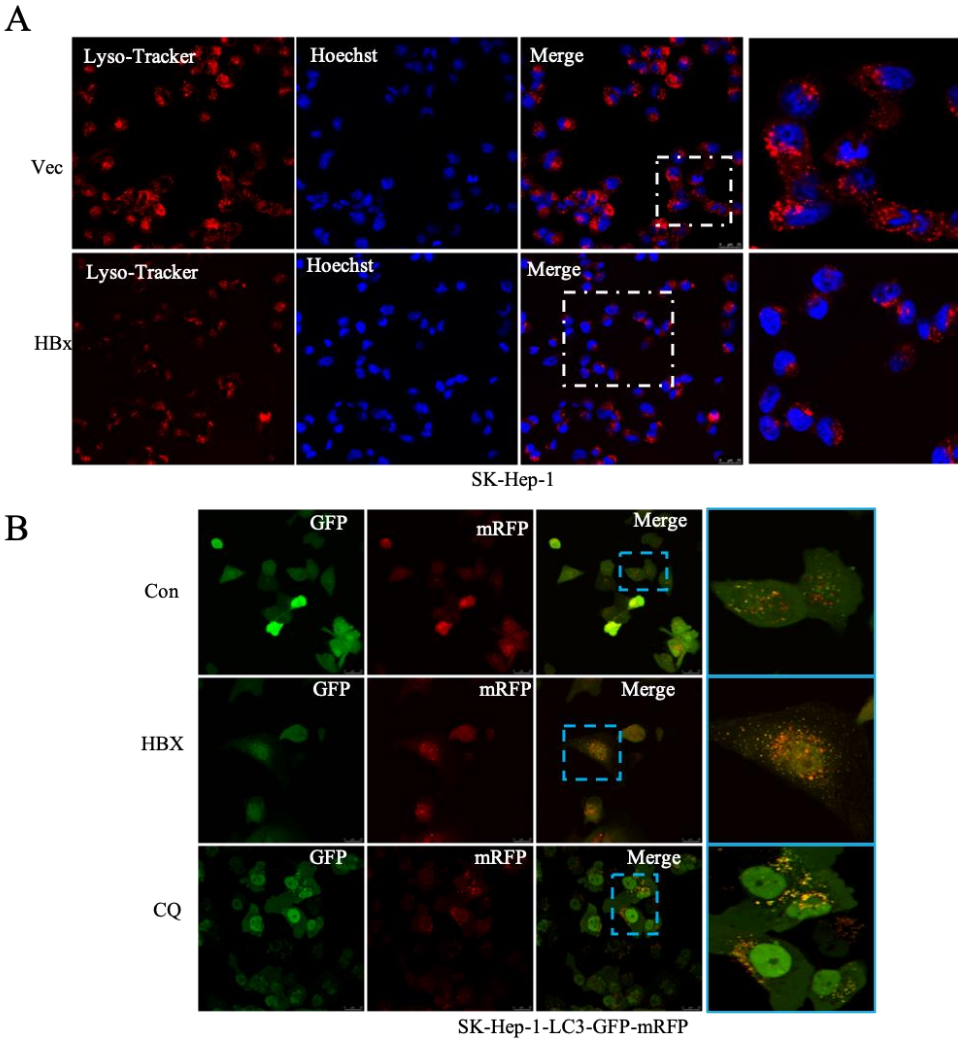

Figure S2. HBx impaired TFEB induced lysosome biogenesis. **(A)** SK-Hep-1 cells expressing vector (Vec) or HBx were treated with 100nM Lyso-Tracker for 1hr. to label the lysosomes and the nucleus were labeled with Hoechst, take photos with confocal microscope. The experiment was repeated three times with five visual fields for statistics. (B**)** SK-Hep-1 cells stable expressing LC3-tandem-GFP-mRFP were expressed with HBx or treated with CQ 50 μm, 12 hr. , take photos with confocal microscope. *** $p<0.001$

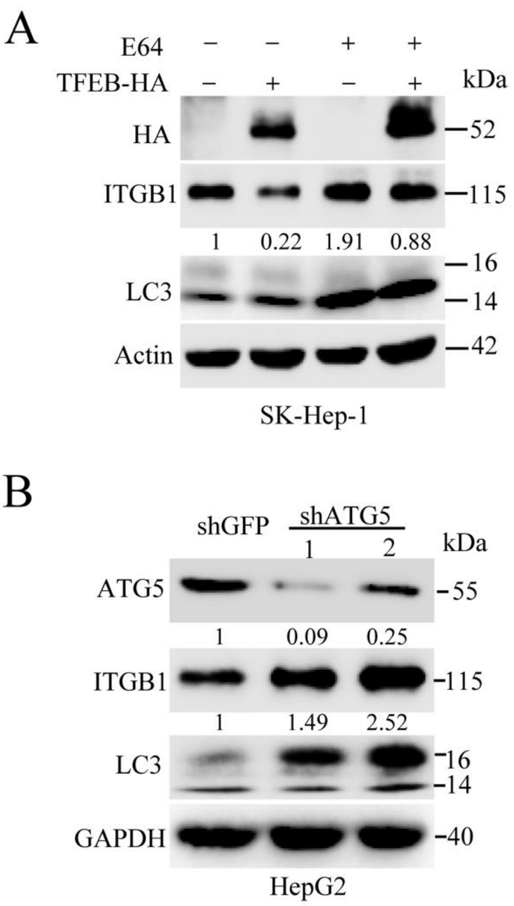

Figure S3. TFEB promoted integrin β1 degradation through auto-lysosomal pathway. **(A)** SK-Hep-1 cells stable expressing TFEB or Vec were treated with 50μM E64 for 24hr., then subjected to Western Blotting analyses. **(B)** HepG2 cells knocking-down of ATG5 were subjected to Western Blotting analyses.

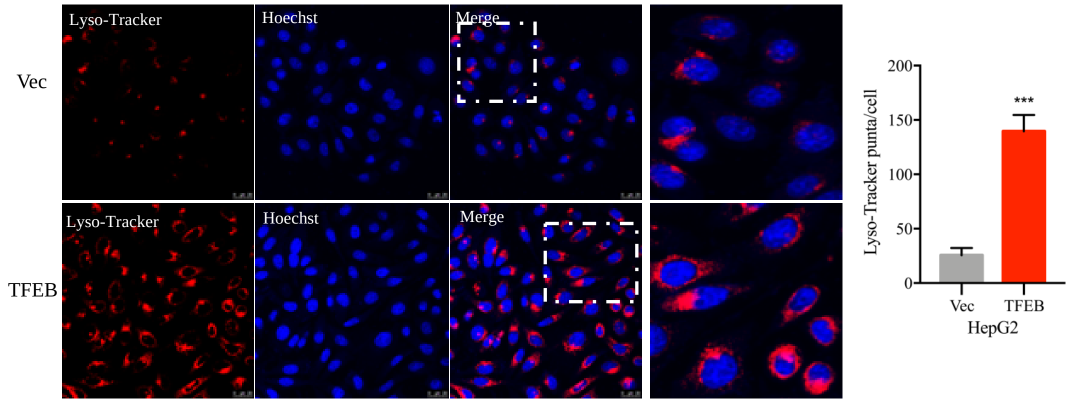

HepG2

Figure S4. TFEB promoted lysosomal biogenesis. **(A)**Stable HepG2 cells expressing vector (Vec) or TFEB were treated with 100 nM Lyso-Tracker for 1hr. to label the lysosomes and the nucleus were labeled with Hoechst, take photos with confocal microscope. The experiment was repeated three times with five visual fields for statistics.； *** $p < 0.001$

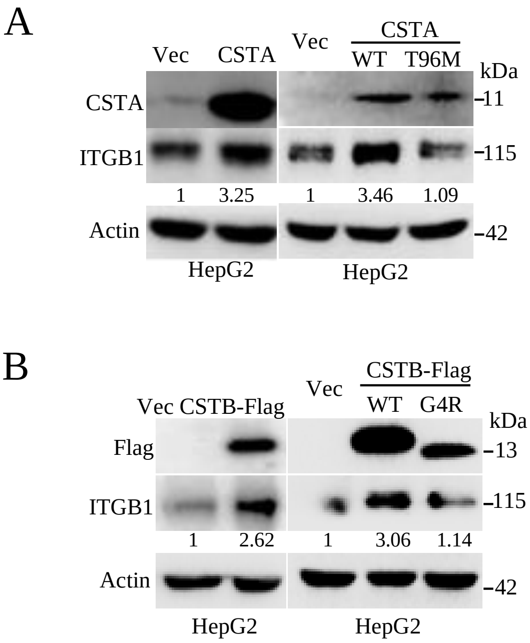

Figure S5. Cellular turnover of ITGB1 was controlled by lysosomal proteinase inhibitors. **(A-B)** HepG2 cells stably expressing CSTA or CSTA^{T96M}(A) or CSTB-Flag or CSTB^{G4R}-Flag (B) were subjected to Western Blotting analyses.

Tabe S1: The sequence information of the primers used for shRNA or variants

Name	Sequence (5'-3')
shTFEB-1-HUM	CCCACTTTGGTGCTAATAGCT
shTFEB-2-HUM	CGATGTCCTTGGCTACATGAA
shCTSL-1-HUM	AAGGCGATGCACAACAGATTA
shCTSL-2-HUM	AAGGCGATGCACAACAGATTA
shATG5-1-HUM	GATTCATGGAATTGAGCCAAT
shATG5-2-HUM	GCAGAACCATACTATTTGCTT
shATG7-1-HUM	GCCTGCTGAGGAGCTCTCCAT
shATG7-2-HUM	CCAGAGAGTTTACCTCTCATT
pLVX-puro-CTSB^{G4R}	F: CCGGAATTCGCCACCATGATGTGCAGGGCGCCC<br>R: GCATCTAGAGTCGCGGGATCCCTAC
pLVX-puro-CTSB^{T96M}	F: CCGGAATTCGCCACCATGATACC<br>R: CGCGGATCCCTACTTATCGTCGTCATCCTTGTAATCAAAGCCCATCAGCTC
